# *CUX2*, *BRAP* and *ALDH2* are associated with metabolic traits in people with excessive alcohol consumption

**DOI:** 10.1038/s41598-020-75199-y

**Published:** 2020-10-22

**Authors:** I-Chun Chen, Po-Hsiu Kuo, Albert C. Yang, Shih-Jen Tsai, Tung-Hsia Liu, Hung-Jen Liu, Tsuo-Hung Lan, Hong-Ming Chen, Huang-Nan Huang, Ren-Hua Chung, Yu-Li Liu

**Affiliations:** 1 Department of Psychiatry, Taichung Veterans General Hospital, Taichung City, Taiwan; 2 Ph.D. Program in Translational Medicine, National Chung Hsing University, Taichung City, Taiwan; 3 Rong Hsing Research Center For Translational Medicine, National Chung Hsing University, Taichung City, Taiwan; 4 Institute of Epidemiology and Preventive Medicine, College of Public Health, National Taiwan University, Taipei, Taiwan; 5 Department of Public Health, College of Public Health, National Taiwan University, Taipei, Taiwan; 6 Division of Interdisciplinary Medicine and Biotechnology, Beth Israel Deaconess Medical Center, Harvard Medical School, Boston, MA USA; 7 Institute of Brain Science, National Yang-Ming University, Taipei, Taiwan; 8 Division of Psychiatry, National Yang-Ming University, Taipei, Taiwan; 9 Department of Psychiatry, Taipei Veterans General Hospital, Taipei, Taiwan; 10 Center for Neuropsychiatric Research, National Health Research Institutes, 35 Keyan Road, Zhunan Town, Miaoli County 35053 Taiwan; 11 Institute of Molecular Biology, National Chung Hsing University, 145 Xingda Road, South District, Taichung City, 402 Taiwan; 12 Faculty of Medicine, National Yang-Ming University, Taipei, Taiwan; 13 Department of Applied Mathematics, Tunghai University, Taichung City, Taiwan; 14 Division of Biostatistics and Bioinformatics, Institute of Population Health Sciences, National Health Research Institutes, Taipei, Taiwan; 15 Graduate Institute of Clinical Medical Science, China Medical University, Taichung City, Taiwan

**Keywords:** Biochemistry, Computational biology and bioinformatics, Molecular biology, Neuroscience, Gastroenterology, Risk factors

## Abstract

Molecular mechanisms that prompt or mitigate excessive alcohol consumption could be partly explained by metabolic shifts. This genome-wide association study aims to identify the susceptibility gene loci for excessive alcohol consumption by jointly measuring weekly alcohol consumption and γ-GT levels. We analysed the Taiwan Biobank data of 18,363 Taiwanese people, including 1945 with excessive alcohol use. We found that one or two copies of the G allele in rs671 (*ALDH2*) increased the risk of excessive alcohol consumption, while one or two copies of the C allele in rs3782886 (*BRAP*) reduced the risk of excessive alcohol consumption. To minimize the influence of extensive regional linkage disequilibrium, we used the ridge regression. The ridge coefficients of rs7398833, rs671 and rs3782886 were unchanged across different values of the shrinkage parameter. The three variants corresponded to posttranscriptional activity, including cut-like homeobox 2 (a protein coded by *CUX2*), Glu504Lys of acetaldehyde dehydrogenase 2 (a protein encoded by *ALDH2*) and Glu4Gly of BRCA1-associated protein (a protein encoded by *BRAP*). We found that Glu504Lys of *ALDH2* and Glu4Gly of *BRAP* are involved in the negative regulation of excessive alcohol consumption. The mechanism underlying the γ-GT-catalytic metabolic reaction in excessive alcohol consumption is associated with *ALDH2*, *BRAP* and *CUX2*. Further study is needed to clarify the roles of *ALDH2*, *BRAP* and *CUX2* in the liver–brain endocrine axis connecting metabolic shifts with excessive alcohol consumption*.*

## Introduction

The recommended level of low-risk alcohol consumption is < 100 g/week^1^. Phenotypes of excessive alcohol consumption are expressed in several forms. Before development of alcohol use disorder, the condition, for example, may initiate with problematic drinking, which has a 2.1% prevalence in Asian countries^2,3^. Excessive alcohol consumption creates a medical and social burden and is associated with alcohol-related liver diseases, public safety incidents, and trauma-related admissions to hospitals.

The genetic architecture of alcohol consumption involves the genetic liability of alcohol use disorder, metabolism, risky behaviors and cognitive phenotypes^4^. The alcohol dehydrogenase 1B (*ADH1B*), alcohol-metabolizing acetaldehyde dehydrogenase 2 (*ALDH2*), β-Klotho (*KLB*), glucokinase regulator (*GCKR*), corticotropin releasing hormone receptor 1 (*CRHR1*), and cell adhesion molecule 2 (*CADM2*) show strong links to drinking behaviours^4–6^. *GCKR* is associated with both alcohol consumption and alcohol use disorder^4^. The role of dopamine receptor D2 subtype (*DRD2*) has been confirmed and replicated in a large-scale genome-wide association study (GWAS)^7^.

Genes that act in pleiotropy across various systems (e.g., cardiovascular, adrenal, pancreatic and central nervous systems) form the genetic picture of excessive alcohol consumption. The alcohol-decreasing allele in *ADH1B* gene was associated with lower odds of coronary heart disease, and those SNPs significantly associated with alcohol consumption were associated with high-density lipoprotein cholesterol levels^8^. The largest study of GWAS on tobacco and alcohol uses involved 1.2 million individuals and uncovered genetic bearing of *ADH1B* and *GCKR*, suggesting that alcohol consumption is influenced by individual differences in one’s ability to process calorie-rich alcoholic beverages^9^. Studies have replicated the *KLB/FGF21* interaction in the putative liver-brain axis^10,11^; and notably, neuronal FGF21 senses metabolic changes in the peripheral tissues, resulting in homeostatic regulation of the liver-brain axis^12^.

Alcohol is chemically bound to hydrophobic amino acids and hydrogen-bonding polar groups of channel proteins^13^, which drive “go pathways” and “stop pathways” in the intracellular level. The “go pathways” are signalling cascades that contribute to the transition from moderate to excessive alcohol intake, including activation of protein kinase A (PKA) and calcium/calmodulin-dependent protein kinase II, whereas the “stop pathways” keep alcohol intake in check, by upregulation of brain-derived neurotropic factor (BDNF) and glial cell line-derived neurotropic factor (GDNF)^14^.

Alcohol Use Disorder Identification Test (AUDIT) makes specific quantitative statements about alcohol consumption versus alcohol use disorder, while Alcohol Use Disorder Identification Test-Consumption (AUDIT-C) measure alcohol consumption. The AUDIT can be applied as a proxy measurement to increase sample sizes in a GWAS on alcohol use disorder^15^. Physiological biomarkers may be used to identify persistent and excessive alcohol consumption. The ethanol intake of excessive drinkers is reflected in the ratio of carbohydrate transferrin to transferrin^16^. The extent of elevated aminotransferase levels in the body is also helpful in detecting alcohol abuse^17^. Asymptomatic patients with alcoholic liver disease present serum levels of γ-glutamyl transpeptidase (γ-GT) doubling that of normal^17^. The γ-GT, which is involved in the metabolism of glutathione, is a major antioxidant in humans, and it is also a common biomarker used in studying alcohol use disorder^18,19^.

It has been suggested in a large-scale GWAS on alcohol consumption^4^ and also other conditions, that to control the effect of population stratification, one may analyze participants according to races and ethnicities. The population of Taiwan comprises 92.6% southern Han Chinese, 4.9% northern Han Chinese, and 1.9% aborigines of Southeast Asian and Austronesian descent^20^. Genetic intermixing between these ethnic groups is rare, resulting in a genetically homogenous Taiwanese population of mostly Han Chinese descent. Our study aims to infer the susceptibility gene loci of excessive alcohol consumption by jointly measuring weekly alcohol consumption and γ-GT levels.

## Results

**Figure 1:**
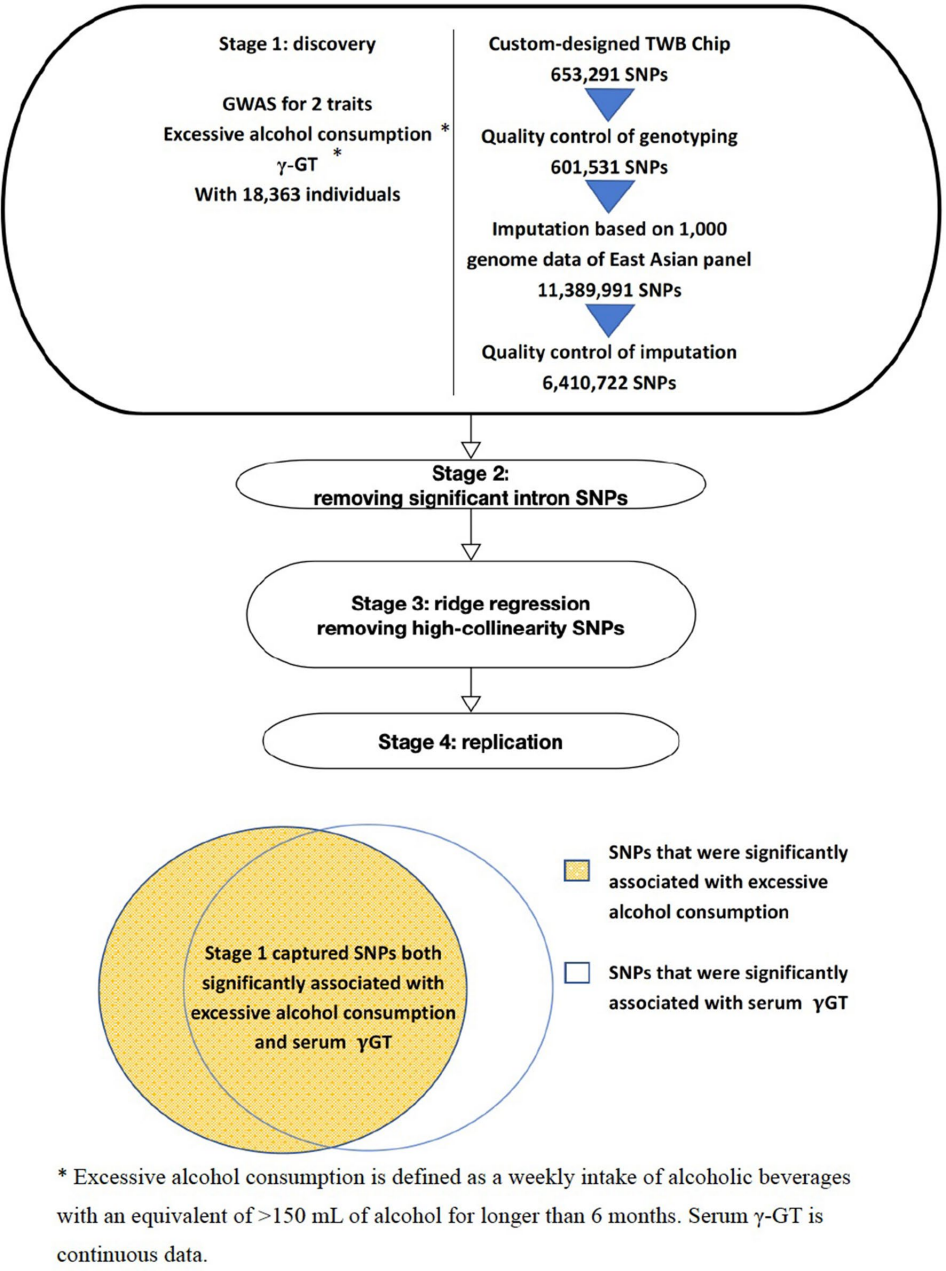
Overall study scheme. GWAS: genome-wide association study, TWB: Taiwan Biobank, SNP: single nucleotide polymorphism.

We retrieved data on the whole-genome genotyping, and also the levels of serum γ-GT and medical history of the 18,363 people whose information had been held in the TWB. Of the participants, 9275 were women. “Excessive alcohol consumption” was defined as a weekly intake of alcoholic beverages with an equivalent of > 150 mL of alcohol for ≥ 6 months. To identify the phenotype of excessive alcohol consumption, we used serum γ-GT as an add-on trait (Fig. 1).

To plot the genetic ancestry of our cohort from Taiwan Biobank (TWB), we used principal component analysis (PCA), and results confirmed a reliable distribution (see Supplementary Fig. S1 online). In this cohort, 1945 participants (10.60%; 87.9% men) had excessive alcohol use (weekly intake of > 150 mL of alcohol for ≥ 6 months) (Table 1, see Supplementary Fig. S2 online). The average serum γ-GT level of those with excessive alcohol use was 46.15 ± 77.08 U/L, higher than those without (23.60 ± 25.71 U/L). Among excessive alcohol users, a significant correlation was found between alcohol consumption and serum γ-GT levels (*p* < 1 × 10^–3^).

**Table 1:** γ-GT, age, and sex for the two groups differentiated by their alcohol consumption.

	Excessive alcohol consumption^a^ (N = 1945)		No excessive alcohol consumption (N = 16,405)	
	N; MEAN (S.D.)	N (%)	N; mean (S.D.)	N (%)
γ-GT^b^ (U/L)	1158; 46.15 (77.08)^c^		9663; 23.60 (25.71)^c^	
Age	1945; 49.55 (10.31)		16,405; 48.72 (11.07)	
**Sex**				
Male		1710 (87.92%)		7369 (44.92%)
Female		235 (12.08%)		9036 (55.08%)

S.D.: Standard deviation.

^a^Excessive alcohol consumption is defined as a weekly intake > 150 mL of alcohol for > 6 months.

^b^γ-GT: γ-glutamyl transpeptidase.

^c^There is a significant difference in serum γ-GT levels between these two groups (*p* < 1 × 10^–3^).

**Figure 2:**
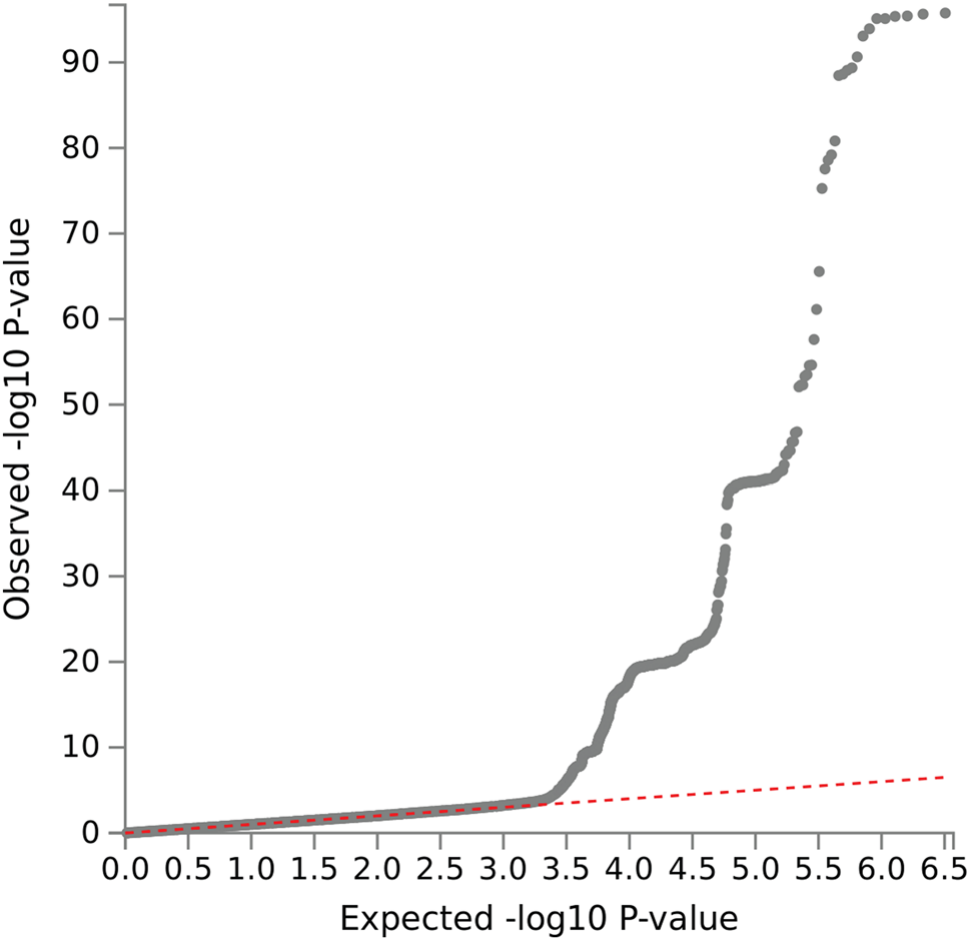
Q–Q plot of the SNP-based test for the drinking variable, adjusted for age, sex, and 10 PCs. Q-Q plot: quantile–quantile plot, SNP: single nucleotide polymorphism, PCs: principal components.

There were 1794 SNPs significantly associated with excessive alcohol use (*p* < 5 × 10^–8^) (see Supplementary Table S1 online). The COJO analysis of GCTA suggests that there were 3 independent signals among these SNPs. LocusZoom plots for the 3 SNPs are shown in Supplementary Fig. S3 online. The plot of log quantile–quantile (Q-Q) *p* values suggested only a few systematic sources of spurious associations (Fig. 2). Because the QQ plot contains a wider range of the observed − log10 *p* values, we further applied LD Score regression (LDSC) to analyze polygenicity and other factors^21^. The estimated LDSC intercept was 1.0083 with a standard error of 0.0056. Furthermore, the genomic inflation factor (*λ*_*GC*_) was also reported by LDSC. The value of *λ*_*GC*_ was estimated to be 1.0043. Both LDSC intercept and *λ*_*GC*_ are close to 1, suggesting no inflation had occurred in our analysis due to confounding factors. The inflation observed in the QQ plot could be driven by a few causal signals as suggested by the COJO analysis and the LocusZoom plots, while many SNPs close to the causal signals are in high linkage disequilibrium (LD) with the causal SNPs. Those SNPs observed corresponded to small *p* values most likely mapped to susceptibility risk loci for excessive alcohol use (Table 2).

**Table 2:** Loci significantly associated with both excessive alcohol consumption and γ-GT.

Gene	Start	End	Minimum *p* value of drinking	Number of overlapping SNPs
*ALDH2*	112204691	112247782	1.73E−96	20
*ACAD10*	112123857	112194903	3.78E−96	31
*BRAP*	112079950	112123790	7.68E−96	24
*HECTD4*	112597992	112819896	1.19E−94	185
*PTPN11*	112856155	112947717	8.45E−94	11
*NAA25*	112464500	112546826	2.27E−91	71
*TRAFD1*	112563305	112591407	8.44E−90	16
*RPH3A*	113008184	113336686	6.35E−80	188
*RPL6*	112842994	112856642	5.46E−76	2
*CUX2*	111471828	111788358	2.76E−66	39
*MYL2*	111348623	111358526	1.05E−43	30
*CCDC63*	111284573	111345339	7.33E−43	65
*ATXN2*	111890018	112037480	2.60E−39	86
*TMEM116*	112369086	112450970	1.35E−29	117
*MAPKAPK5*	112279782	112334343	1.36E−25	100
*SH2B3*	111843752	111889427	9.87E−25	18
*ERP29*	112451120	112461255	3.54E−20	9
*IFT81*	110562140	110656602	2.47E−19	3

Adjusted for age, sex, and 10 principal components. *ALDH2*: aldehyde dehydrogenase 2, *ACAD10*: acyl-coenzyme A dehydrogenase family, member 10, *BRAP*: breast cancer 1-associated protein, *HECTD4*: HECT domain E3 ubiquitin protein ligase 4, *PTPN11*: protein tyrosine phosphatase non-receptor type 11, *NAA25*: N(alpha)-acetyltransferase 25, *TRAFD1*: TRAF-type zinc finger domain containing 1, *RPH3A*: rabphilin 3A, *RPL6*: ribosomal protein L6, *CUX2*: cut-like homeobox 2, *MYL2*: myosin, light polypeptide 2, *ATXN2*: ataxin 2, *CCDC63*: coiled-coil domain containing 63, *TMEM116*: transmembrane protein 116, *MAPKAPK5*: MAP kinase activated protein kinase 5, *SH2B3*: SH2B adaptor protein 3, *ERP29*: endoplasmic reticulum protein 29, *IFT81*: intraflagellar transport 81.

**Figure 3:**
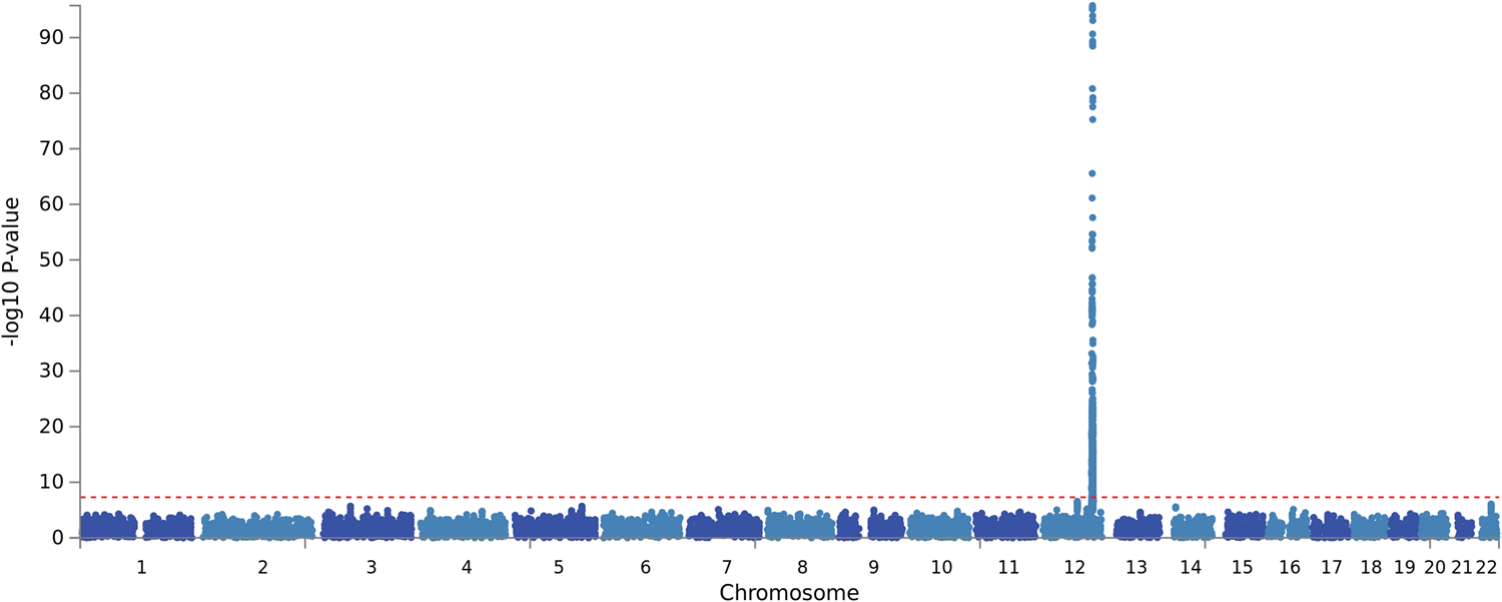
Manhattan plot of SNP-based test for the drinking variable, adjusted for age, sex, and 10 PCs. SNP: single nucleotide polymorphism, PCs: principal components.

We captured 1015 SNPs that were both significantly associated with excessive alcohol use and with γ-GT (FDR < 0.05) (Fig. 1; Table 2). These 1015 significant SNPs aggregated on chromosome 12 (Fig. 3). They were identified within a region of approximately 3.7 million bases located between *TRPV4* and *SDS* (chr12: 110238596–113944048) (see Supplementary Table S1 online). The strongest signal appeared at rs671 (*ALDH2*), where the codon change from the G allele to the A allele creates a missense variant and represents the translation from glutamic acid to lysine in the sequence (see Supplementary Table S2 online). The SNP rs671 is in strong LD with rs4646776 (LD *r*^2^ = 0.998), one of the three independent SNPs identified by the COJO analysis, suggesting that they are in the same LD block.

**Figure 4:**
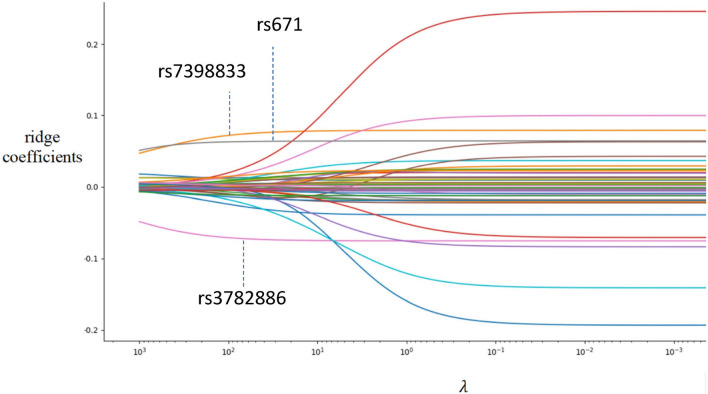
The X axis represents the weights, which are the ridge coefficients ($\hat{\beta}^{ridge}$) corresponding to 48 SNPs. The Y axis represents the shrinkage parameter *λ*, which controls the size of the coefficients and the amount of regularisation. Curves of the ridge coefficients as a function of regularisation. Note that rs7398833, rs671 and rs3782886 have $\hat{\beta}^{ridge}$ values that are maximal values away from zero and remain constant across different *λ* values. Those curves sharply alienated from X axis indicate dependent signals of linkage disequilibrium.

To find the possible causal variants of excessive alcohol consumption within this region, we further identified their coding variants. We used coding-synonymous SNPs, 5′ untranslated region SNPs, missense SNPs, non-coding RNA elements in the 3′ untranslated regions, cds-indels, and frameshift mutations to obtain 48 significant SNPs. Among these 48 SNPs, rs7398833 (*CUX2*), rs671 (*ALDH2*) and rs3782886 (*BRAP*) had ridge coefficients, $\hat{\beta}^{ridge}$, that remained unchanged across different values of the shrinkage parameter $\lambda$ (Fig. 4). The rs7398833 (*CUX2*) is located in the 3′ untranslated region (3′-UTR), where it post-translationally manipulates the stability of CUX2. The coding change from T to C allele at rs3782886 (*BRAP*) creates a missense variant, which leads to a coding change from glutamic acid to glycine in the translation of BRCA1-associated protein isoform 4.

In our participants, a strong LD was found between rs671 and rs3782886 (r^2^ = 0.98) (see Supplementary Fig. S4 online). A significant haplotype was therefore associated with excessive alcohol consumption and it was comprised of both rs671 and rs3782886. The presence of a haplotype carrying the G allele of rs671 and T allele of rs3782886 (haplotype GT) showed an odds ratio (OR) of 2.49 (95% confidence interval CI 2.27–2.72) for excessive alcohol consumption, whereas a haplotype carrying A allele of rs671 and C allele of rs3782886 (haplotype AC) had an odds ratio (OR) of 0.4 (95% CI 0.37–0.44). Comparing levels of γ-GT between carriers with haplotype GT and those with haplotype AC, we found a differential increment of 2.42 ± 0.53 U/L (*p* = 4.92 × 10^–6^).

We performed conditional analysis to identify independent signals between rs671 (*ALDH2*) and rs3782886 (*BRAP*). We compared using a partial F-test, three models each with rs671 (*ALDH2*), with rs3782886 (*BRAP*), then with both rs671 (*ALDH2*) and rs3782886 (*BRAP*). The regression coefficients estimated were: rs671 (*ALDH2*), rs3782886 (*BRAP*) and rs7398833 (*CUX2*) were estimated as 3.54 (95% CI 1.06, 6.02) for model rs671 (*ALDH2*), − 1.98 (95% CI − 3.38, − 1.59) for model rs3782886 (*BRAP*) and − 3.64 (95% CI − 13.51, 6.24) for model rs7398833 (*CUX2*). Regarding the direction of effects, one or two copies of G allele in rs671 (*ALDH2*) increased the risk of excessive alcohol consumption, while one or two copies of C allele in rs3782886 (*BRAP*) reduced the risk of excessive alcohol consumption. We found that the model that included both rs671 (*ALDH2*) and rs3782886 (*BRAP*) was significantly better with a significantly lower sum of squared error (*p* < 0.01) (see Supplementary Table S3 online).

**Figure 5:**
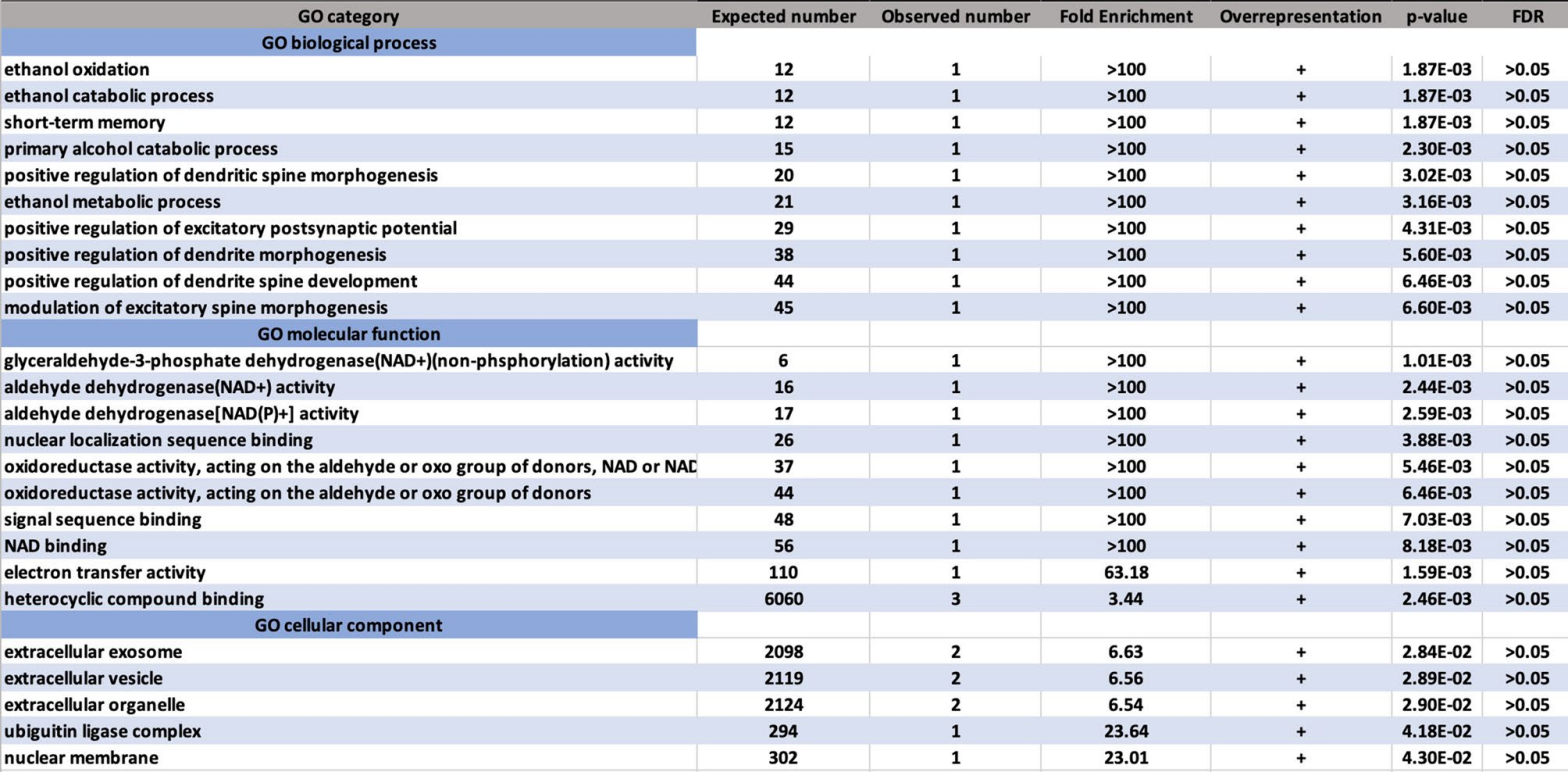
Results of gene set-based analysis for metabolic traits among people with excessive alcohol consumption. First column: The Gene Ontology (GO) category. Second column: The number of genes expected in this category. Third column: The observed number of genes that map to this GO category. Forth column: Fold Enrichment is the observed number divided by the expected number. If it is greater than 1, it indicates that the category is overrepresented. Fifth column: A plus sign indicates overrepresentation of this GO category. Sixth column: Cutoff is 0.05. Seventh column: The probability that the number of genes observed in this category occurred by chance.

We also performed gene set-based analyses using gene sets including *ALDH2*, *BRAP* and *CUX2*. The gene set-based analyses for metabolic traits among excessive alcohol drinkers generated results in Fig. 5. None of these genes showed significant fold enrichment (FDR > 0.05).

For access to replication study, we compared of results of this GWAS with the publicly available database on the GWAS ATLAS resource (https://atlas.ctglab.nl/)^22^, a post-GWAS fine-mapping study in individuals of Korean descent (459 with alcohol dependence, 455 controls) and a trans-population GWAS meta-analysis of AUDIT-C (N = 274,424)^7,23^. A total of 45 GWASs were derived from the GWAS ALTAS resource (average sample size = 132,522). The multiple GWAS comparisons in the GWAS ATLAS resource grouped physically overlapping risk loci, and identified risk loci from 111599617 to 111705565 on chromosome 12. This region mapped to *BRAP* and *ALDH2*. The post-GWAS fine-mapping study on participants of Korean descent showed a genetic correlation between rs3782886 (*BRAP)* and alcohol dependence (*p* = 9.94 × 10^−31^), with the minor homozygote associating with lesser risk of alcohol consumption^23^. This adheres to our finding that one or two copies of C allele in rs3782886 (*BRAP*) reduced the risk of excessive alcohol consumption. A trans-population GWAS meta-analysis of AUDIT, including 1410 cases of excessive alcohol consumption in the East Asian subgroup of the 274,424 individuals, found a risk locus in *BRAP*^7^. We found that rs3782886 (*BRAP*) and rs671 (*ALDH2*) were associated with pleiotropy across various systems including metabolic conditions (see Supplementary Fig. S5 online). These results do not manifest a replication of the initial findings, but it suggests that both *BRAP* and *ALDH2* influence metabolic traits^22^.

## Discussion

Our main finding is that in excessive alcohol consumption, the γ-GT-catalytic reaction is associated with *ALDH2*, *BRAP* and *CUX2*. Both the A allele in rs671 (*ALDH2*), and the C allele in rs3782886 (*BRAP*) lowered risks of excessive alcohol consumption. These gene products acted as negative regulators on excessive alcohol consumption.

Our GWAS has several strengths. First, we developed a new approach for evaluating intermarker linkage disequilibrium. Conducting ridge regression led to the identification of significant SNPs. For complex traits like excessive alcohol consumption, strategies to elucidate polygenicity should be considered. Our strategy to tackle the polygenicity and linkage disequilibrium is the use of ridge regression, which has proven to efficiently identify genetic markers of complex genetic disorders^24–26^. Like linkage disequilibrium score regression, ridge regression can help resolve SNPs in strong linkage disequilibrium^24^. Second, we captured SNPs that are significantly associated with both excessive alcohol use and γ-GT. Diagnostic bias was reduced by exhibiting persistent phenotypes with higher alcohol consumption. Third, our use of a nationwide biobank provided statistical power of our tests greater than those of previous studies^27^.

Our analysis of TWB revealed that 71% of participants carried the G allele at rs671 and 29% carried the A allele. As for rs3782886, 71% of subjects carried the T allele and 29% carried the C allele. In other East Asian populations, at rs671 83% of individuals have the G allele, and 17% have the A allele. In the American, African, European, and South Asian populations, in contrast, this frequency is 100% for the G allele and 0% for the A allele. For East Asian populations, the allele frequency at rs3782886 was 83% for the T allele and 17% for the C allele. However, for all the other population groups, the frequency is 100% for the T allele and 0% for the C allele. The strong linkage disequilibrium between rs671 and rs3782886 (r^2^ = 0.98) as well as the higher proportion of haplotype AC in East Asian populations are the evidence for a race-specific haplotype.

The association of rs3782886 with excessive alcohol use should not be neglected simply due to high linkage disequilibrium with rs671, a well-documented single nucleotide variant encoding the alcohol-metabolism enzyme^28–30^. The reasons against such negligence are as follows.

First, *BRAP* is associated with a risk of myocardial infarction and a phenotype of metabolic traits in Asian populations^31,32^. *BRAP* is a risk locus for metabolic syndrome^32^. Metabolites associated with alcohol consumption are primarily involved in amino and fatty acid metabolism^33,34^. During ethanol metabolism as well as NADH and acetyl-CoA build up, more acetyl-CoA generate more malonyl-CoA. For fatty acid metabolism, that leads to inhibition of catabolism and activation of synthesis. Studies of *Caenorhabditis elegans* have demonstrated that *BRAP2* (BRA*P* homolog) regulates the expression of proteins involved in lipid synthesis^35^. During persistent and excessive alcohol consumption, it is clinically implicated to elucidate the mechanisms between *BRAP* and metabolism of amino acid, and fatty acid.

Second, *BRAP* is involved in cerebral cortical neurogenesis^36,37^. For neural progenitor cells, cell signalling during the G1 phase of the cell cycle requires *BRAP*^37^. *BRAP* regulates at the cellular level MAP kinase pathways and the ubiquitin system^38^, which likely controls the cascade of protein turnover during neuronal differentiation. Given that *BRAP* is involved in cell differentiation of the central nervous system, its involvement in mechanisms of neurobiological changes during excessive alcohol consumption should be further explored.

Third, we argue that *BRAP* plays a role in the regulation of reactive oxygen species (ROS) during excessive alcohol consumption^39^. Both alcohol metabolism by CYP2E1 and the reoxidation of NADH via the electron transport chain in the mitochondria generate more ROS^40^. The BRAP/nuclear factor erythroid 2-related factor (Nrf2) signalling cascade responses to oxidative stress^35^, suggesting BRAP regulates ROS during excessive alcohol consumption.

In European populations, other consistently replicated hits in GWASs of alcohol consumption include *KLB*, *FGF21*, and *GCKR*, which are also involved in metabolism. But these hits were not identified in our present study. Plausible explanations of the discrepancy are as follows. First, particularly in East Asians, *BRAP* gene plays the major role in excessive alcohol consumption trait*.* Second, the liver–brain endocrine axis for homeostatic regulation responds to excessive alcohol consumption via FGF21^11,34^, of which both KLB and Nrf2 are substrates closely affected by the nature of diet and food preference^12,41^. It remains unclear as to how BRAP/Nrf2 signalling links to energy use and nutrient use regarding metabolism. Functional analysis is required to determine the role of BRAP/Nrf2 signalling in the liver-brain endocrine axis during the metabolic shift of excessive alcohol consumption.

Here, we reported the novel locus rs7398833 (*CUX2*), which is a 3′-UTR variant that functionally locks or releases the poly-A tail^42^. This function likely maintains the stability of the CUX2 protein and subcellularly localizes the CUX2 protein^42^. Second, *CUX2* is expressed mostly in the brain and is involved in neuronal differentiation in the cortex, specifically acting at the progenitors of GABAergic or dopaminergic neurons^43^. Alcohol is a ligand for both GABAergic and dopaminergic receptors. Further studies to verify the genetic correlation between rs7398833 (*CUX2*) and excessive alcohol consumption are necessary.

We selected loci that were associated with excessive alcohol consumption and elevated levels of γ-GT. The average levels of serum γ-GT, at 46.15 ± 77.08 U/L, fell within the range of those of excessive alcohol users (n = 1945) and are higher than the average level of all 18,363 participants (26.01 ± 35.69 U/L). The high standard deviation of γ-GT levels of the participants with excessive alcohol consumption in our study could reflect asymptomatic patients with alcohol-induced hepatitis^17^.

Alterations in the metabolic profiles of excessive alcohol drinkers involve vastly different systems, such as carbohydrates, lipids, and proteins. To move a step closer to the metabolic traits of people with excessive alcohol consumption, we may need to study targets other than γ-GT. Nonetheless, γ-GT catabolises biliary glutathione and expands the pool of amino acid precursors required for conjugation (glycine [directly] and taurine [through cysteine oxidation]), thus implicating the metabolism of amino acids^44^. Additionally, γ-GT represents the impact of metabolic disease on vascular injury and atherosclerosis^45,46^. In this aspect, our study showed that mechanisms underlying the γ-GT catalytic metabolic reaction among people with excessive alcohol consumption are associated with *ALDH2*, *BRAP* and *CUX2*.

Considering the impact of socioeconomic backgrounds, the living locations, income and education levels were incorporated in measurement of our study. Information of education levels had 0.08% missing data. The income information had 54.3% missing data, and interpretation subject to the lack of thereof. In population-based study, voluntary participation tends to attract individuals with higher education levels and socioeconomic status, as well as lower levels of problem drinking^4^. This trend complemented our study.

Our study has several limitations. First, we excluded significant intronic SNPs and used only significant exonic SNPs. The reason of why we excluded intronic variants was due to the limited sample size. The intronic signals that might be involved in alternative splicing and gene expression were therefore overlooked^43^. As a result, intronic variants that convey a risk of excessive alcohol consumption were likely to be missed. Second, we defined “excessive alcohol consumption” according to the criterion of a weekly intake of > 150 mL of alcohol for > 6 months. The types of beverages consumed were unclear. Low-risk alcohol use of < 100 g/week is equivalent to 7.1 cans of beer (350 mL each, 5% alcohol content) or 1.3 bottles of wine (750 mL, 13%). Our definition of excessive alcohol consumption was stricter than that employed in the literature. However, in the Taiwan Biobank one cannot identify how many of the excessive users had an alcohol use disorder diagnosis. Third, out of 18,363 Taiwanese subjects, 1945 (~ 10%) were defined as cases, and 16,418 participants (~ 90%) were defined as controls in this case–control study. In addition, there was a sex imbalance in this sample. We addressed the limitation of case–control imbalance. In future work, SAIGE (Scalable and Accurate Implementation of Generalized mixed model) could be used to account for sample imbalance^47^. Nonetheless, the PCA plot for the genetic ancestry of this TWB cohort revealed that the distribution had no obvious deviation (see Supplementary Fig. S1 online). Fourth, the majority of individuals from eastern Taiwan and the outlying islands live in rural townships. Supplementary Fig. S6 online shows that the prevalence of excessive alcohol consumption is likely to be different among individuals from northern, central, southern and eastern Taiwan. Those on the outlying islands had higher frequencies of excessive alcohol consumption. Owing to the small sample size from the outlying islands, we did not correct these islanders. Lastly, our findings did not provide directionality of causality (metabolism vs. alcoholism). One way to clarify this issue is to use Mendelian randomisations in future studies.

In conclusion, we developed an alternative strategy for overcoming extensive regional linkage disequilibrium. We uncovered Glu504Lys of *ALDH2* and Glu4Gly of *BRAP*, which are involved in the negative regulation of excessive alcohol consumption. The mechanism underlying the γ-GT catalytic metabolic reaction in excessive alcohol consumption is associated with *ALDH2*, *BRAP* and *CUX2*. Further studies are needed to determine the roles of *ALDH2*, *BRAP* and *CUX2* in the liver-brain endocrine axis upon the metabolic shift with excessive alcohol consumption*.*

## Methods

### Study participants

Data were taken from the TWB, which were random samples of Taiwanese people aged 30 to 70 years old with no history of cancer. Information analyzed was related to genomic data and lifestyle^48,49^. Lifestyle factors included current tobacco use and cigarette smoking, weekly exercise activity of ≥ 3 times, each ≥ 30 min. We measured medical history containing the following conditions: gout, hypertension, hyperlipidaemia, stroke, diabetes mellitus, peptic ulcer, irritable bowel syndrome, migraine, gastric-oesophageal reflex syndrome, depressive disease, bipolar disorder, and schizophrenia. Using posters, brochures, websites, and audio and video media, we recruited TWB participants from 27 outreach centres in the rural and urban townships in Taiwan (see Supplementary Fig. S6 online). All participants signed informed consent forms. This study was approved by the Ethics Review Committees of National Taiwan University Hospital (project number: 201506095RINC).

### Genotyping

In the TWB, whole-genome genotyping was conducted on DNA extracted from blood samples using a QIAamp DNA blood kit, according to the manufacturer’s instructions (Qiagen, Valencia, CA, USA). The qualitative information of the extracted genomic DNA was visualised using agarose gel electrophoresis, and quantitative properties were measured by spectrophotometry. Samples were genotyped with a custom-designed Affymetrix Axiom Genome-Wide Array Plate, which contained 653,291 SNPs. To reach genotyping call-rate of 0.95, SNP and sample quality control thresholds were used in PLINK, a whole-genome data analysis toolset (MIND > 0.05). The identity state was set at 0.4 for each pair of individuals based on the average proportion of alleles shared at the genotyped SNPs. Those SNPs not following the Hardy–Weinberg equilibrium (with cut-off *p* > 1 × 10^–6^) or rare variants with minor allele frequencies (< 1 × 10^–3^) were pruned. In total, 601,531 SNPs remained after the exclusion. Imputation was conducted with the Michigan Imputation Server (https://imputationserver.sph.umich.edu) using 1000G phase 3 v 5 as a reference panel. Eagle v 2.3 was used for phasing, and the EAS population was used for quality control. We imputed 11,389,991 variants of the TWB data based on the East Asian panel of the 1000 Genomes dataset. For imputation quality control, the criteria considered were an imputation quality score of > 0.8 and minor allele frequency of > 0.01. Finally, 6,410,722 variants successfully passed the two quality control stages.

### Statistical analyses

Based on information related to lifestyle, medical history, and the genotypes of 6,410,722 SNPs, we used the principal component analysis to extract 10 principal components for modelling the data. Multivariate logistic regression was used to calculate the odds ratio and *p* value for each SNP, and the model comprised of age, sex, and 10 principle components. We used the additive model to determine genotype risks. The false detection rate (FDR) was calculated to overcome effects of multiple tests. To determine the number of independent signals, the cut-off of FDR is less than 0.05.

Intermarker linkage disequilibrium is possibly caused by distance proximity and the coexpression of genes. If *n* is the number of significant SNPs, there are $C_{2}^{n}$ possible pairs with intermarker linkage disequilibrium. The ordinary least squares approach results in hypercollinearity when a full set of significant SNPs is included in the multivariate regression model. To solve the hypercollinearity problem, we used the ridge regression. Ridge regression minimises a penalty-augmented loss function and obtains the optimisation parameters $\hat{\beta}^{ridge}$.

$$\hat{\beta}^{ridge} = \mathop {argmin}\limits_{{\beta \in R}} \left\| {y - X\beta } \right\|_{2}^{2} + \lambda \left\| \beta \right\|_{2}^{2} ,$$

where $\left\| \beta \right\|_{2} = \sqrt {\beta_{0}^{2} + \beta_{1}^{2} + \cdots + \beta_{p}^{2} }$ and $\lambda$ is the shrinkage parameter that controls the size of coefficients and amount of regularisation. As $\lambda$ approaches zero, the least square solutions are obtained; as $\lambda$ approaches infinity, the ridge coefficients $\hat{\beta}^{ridge} = 0$ are obtained. The result is a constant (intercept-only) model. We selected the SNPs for which $\hat{\beta}^{ridge}$ was stable across different $\lambda$ values.

Statistical analyses were conducted using R, Python open-source programming languages, FUMA GWAS (https://fuma.ctglab.nl/), LDSC software (https://github.com/bulik/ldsc), Plink version 1.90, the Multiple GWAS comparison and PheWAS of the GWAS ATLAS resource (https://atlas.ctglab.nl/), HAPLOVIEW version 4.2, and standard SAS software.

### Gene-set based analysis

To map the most significant genes to particular clusters of biological mechanisms, we conducted gene list analysis. The Gene Ontology (GO) terms were used for functional annotation. We performed gene-list analysis by using PANTHER software and tools^50^. The list of significant genes was uploaded directly on the homepage of the GO website (geneontology.org/docs/go-enrichment-analysis). Hypergeometric distribution was applied to test whether the overrepresentation of a GO term occurred significantly more often than chance. Hypergeometric distribution and binomial test were applied to test whether the overrepresentation of a GO term occurred significantly more often than chance. Cut-off of *p* value is < 0.05. Fold enrichment was defined as the number of significant genes in the list divided by the expected number of genes in a particular GO category^50^.

### Ethics approval

The study abided the Declaration of Helsinki. This study was approved by the Ethics Review Committees of National Taiwan University Hospital (project number: 201506095RINC).

## Supplementary information

Supplementary Information.

## Data Availability

The raw data supporting the conclusion of this article will be made available by the authors, without undue reservation, to any qualified researchers.
