# Supplementary material for: CUX2, BRAP and ALDH2 are associated with metabolic traits in people with excessive alcohol consumption
Source: Sci Rep. 2020 Oct 22;10:18118. doi: 10.1038/s41598-020-75199-y (PMC7583246; doi:10.1038/s41598-020-75199-y)
Supplement: Supplementary file 1 — Supplementary Information. [file 41598_2020_75199_MOESM1_ESM.docx]

***CUX2*, *BRAP* and *ALDH2* are associated with metabolic traits in people with excessive alcohol consumption**

I-Chun Chen^1,2,3^, Po-Hsiu Kuo^4,5^, Albert C. Yang^6,7^, Shih-Jen Tsai^7,8,9^, Tung-Hsia Liu^10^, Hung-Jen Liu^3,11*^, Tsuo-Hung Lan^1,12^, Hong-Ming Chen^13^, Huang-Nan Huang^13^, Ren-Hua Chung^14^, Yu-Li Liu^10,15,*^

**Supplementary Fig. S1.** The PCA plot for the genetic ancestry of this TWB cohort. Each sample was located on the PCA plot. The two axes of PCA plot showed no population structure introduced by population differences.


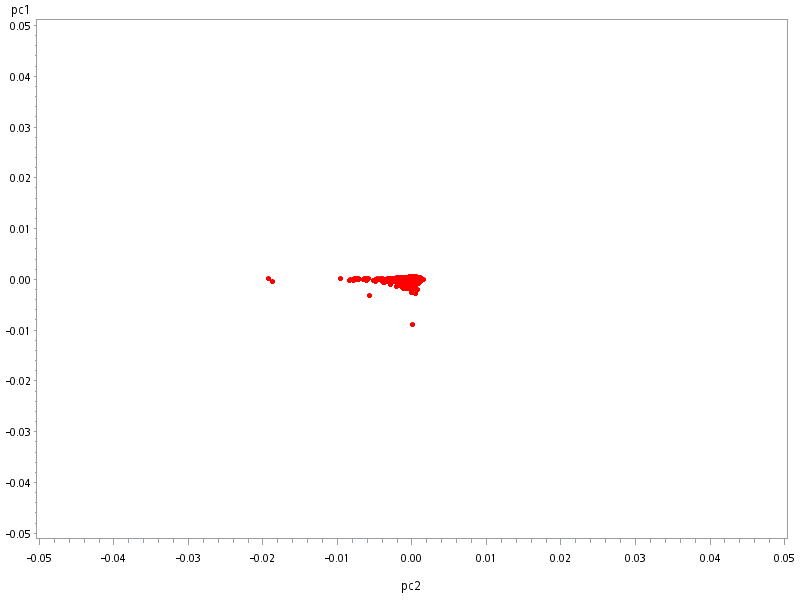


**Supplementary Fig. S2.** The distribution of excessive alcohol consumption and serum γ-GT levels among 18363 subjects in the TWB. *The average serum level of γ-GT is 26.01 U/L, and the S.D. is 35.69 U/L.


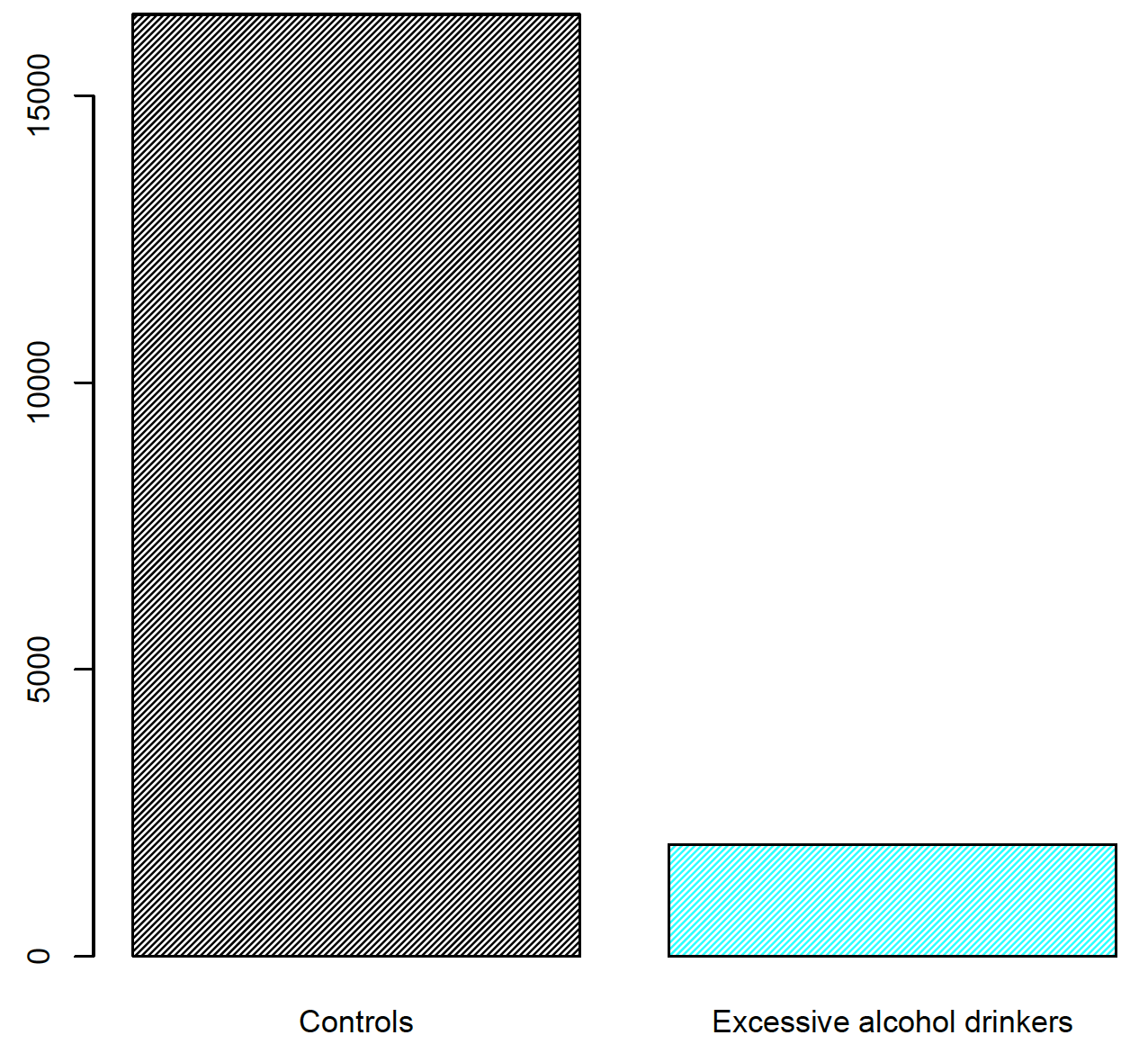


Number of subjects


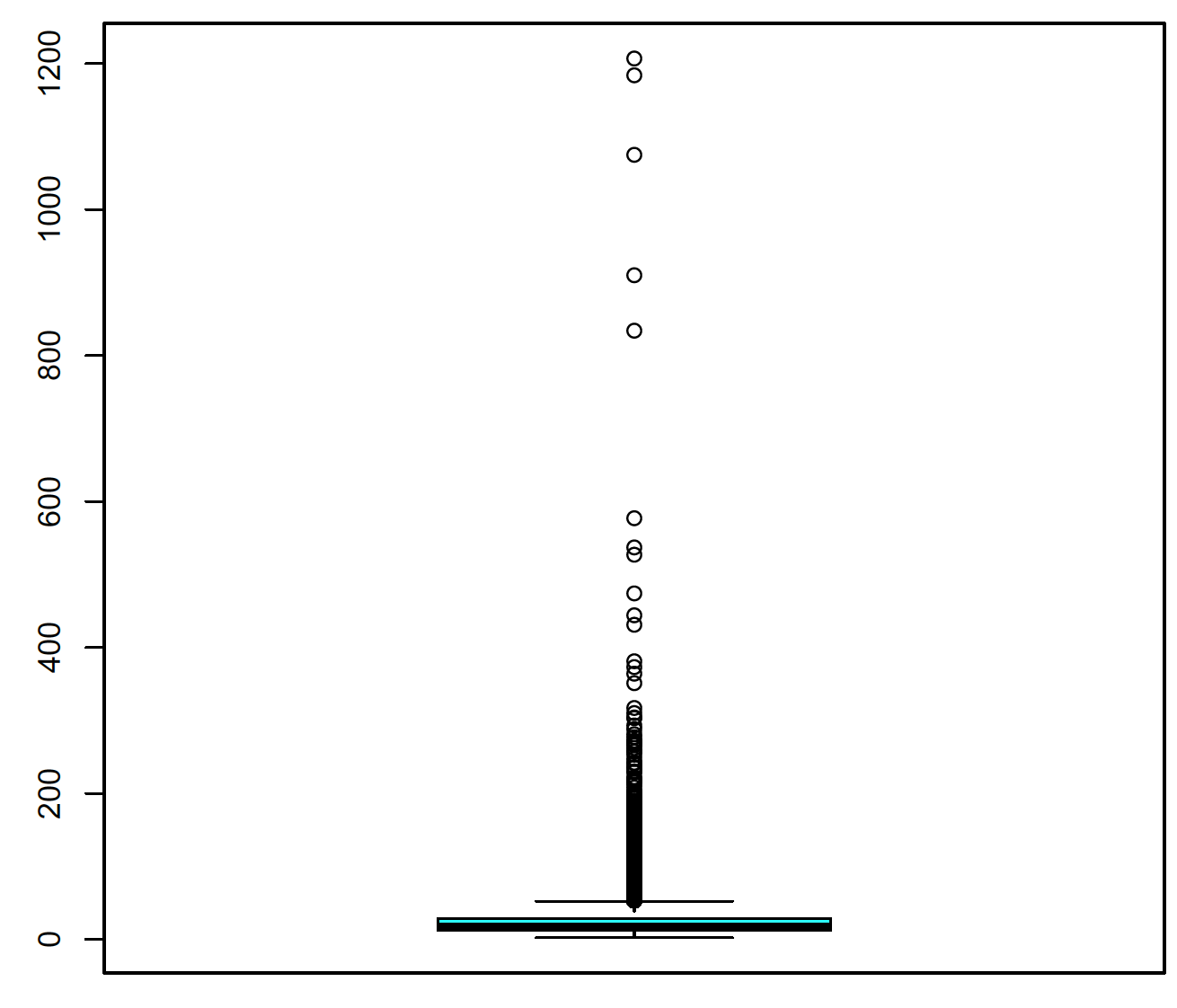


Serum level of

γ-GT*

**Supplementary Fig. S3.** The regional visualization of association plot of LocusZoom in the 3 independent signals ((a) rs3803167, (b) rs4646776 and (c) rs2189271) selected by the COJO function in GCTA. The lead SNP was labelled with purple solid circle.

(a)


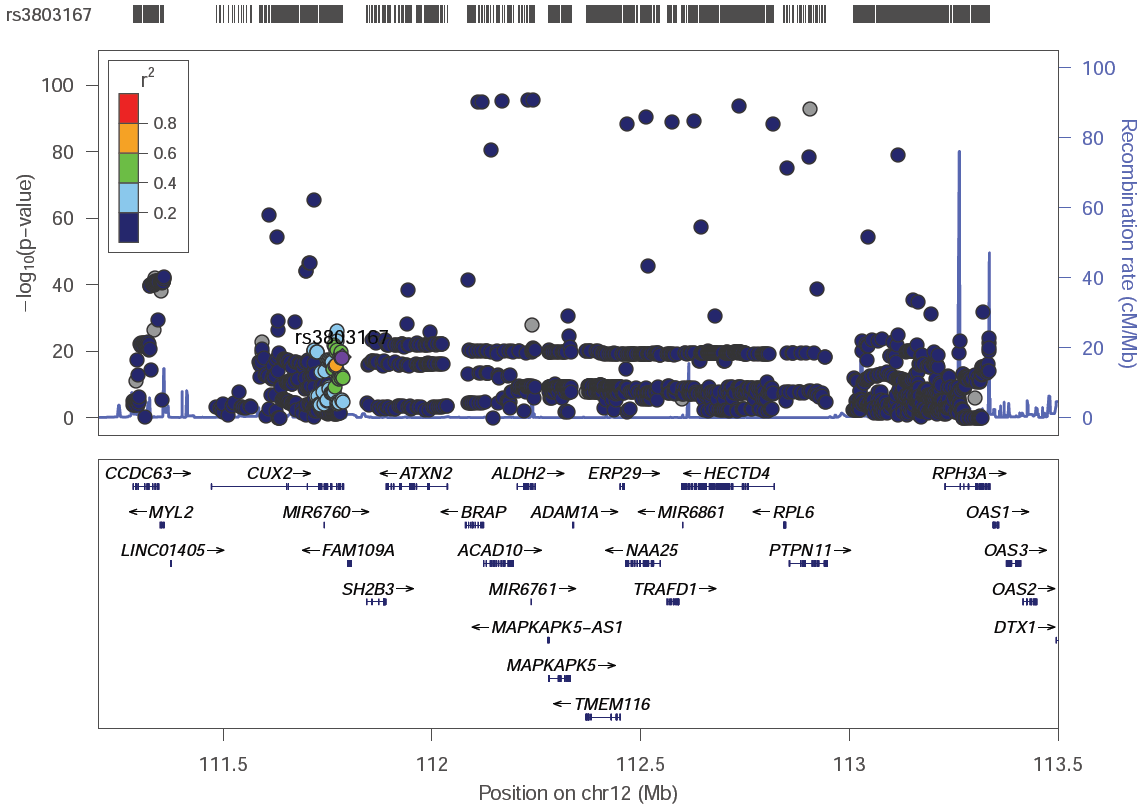


(b)


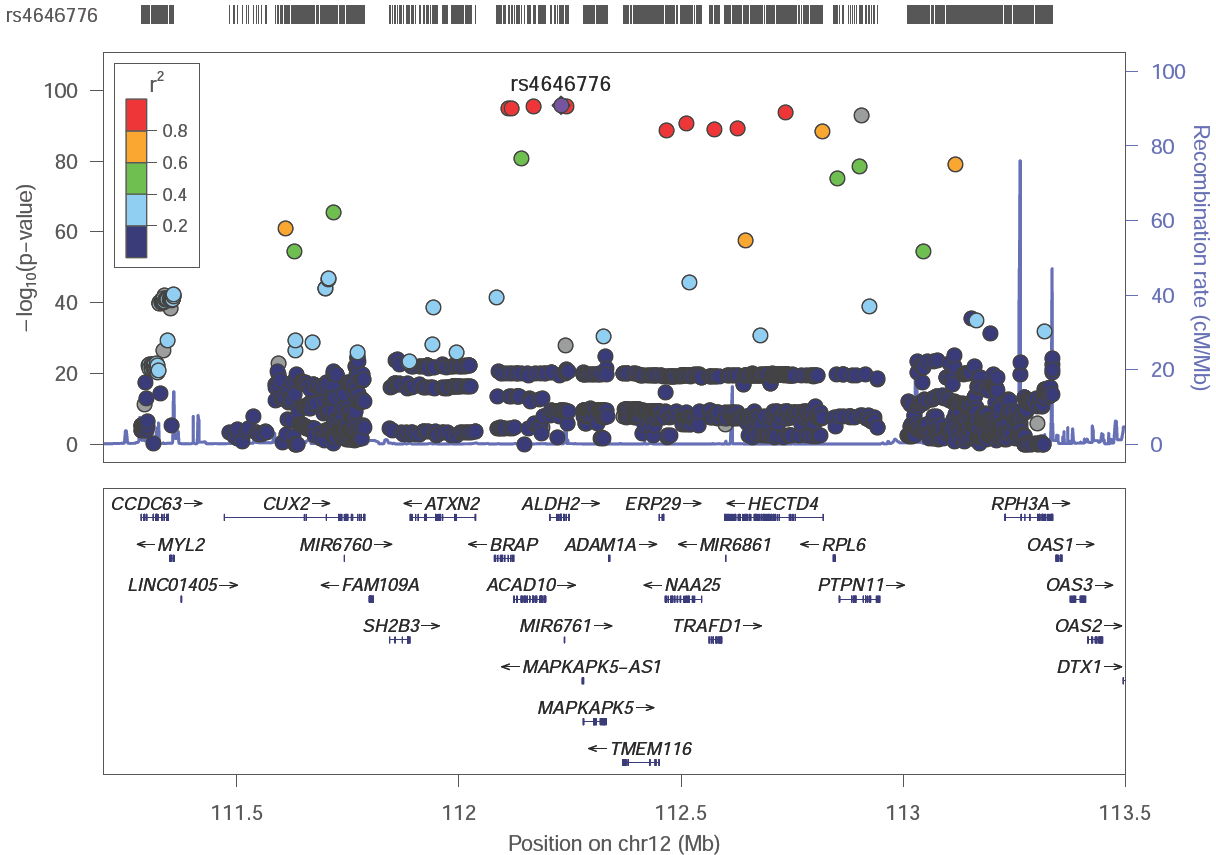


(c)


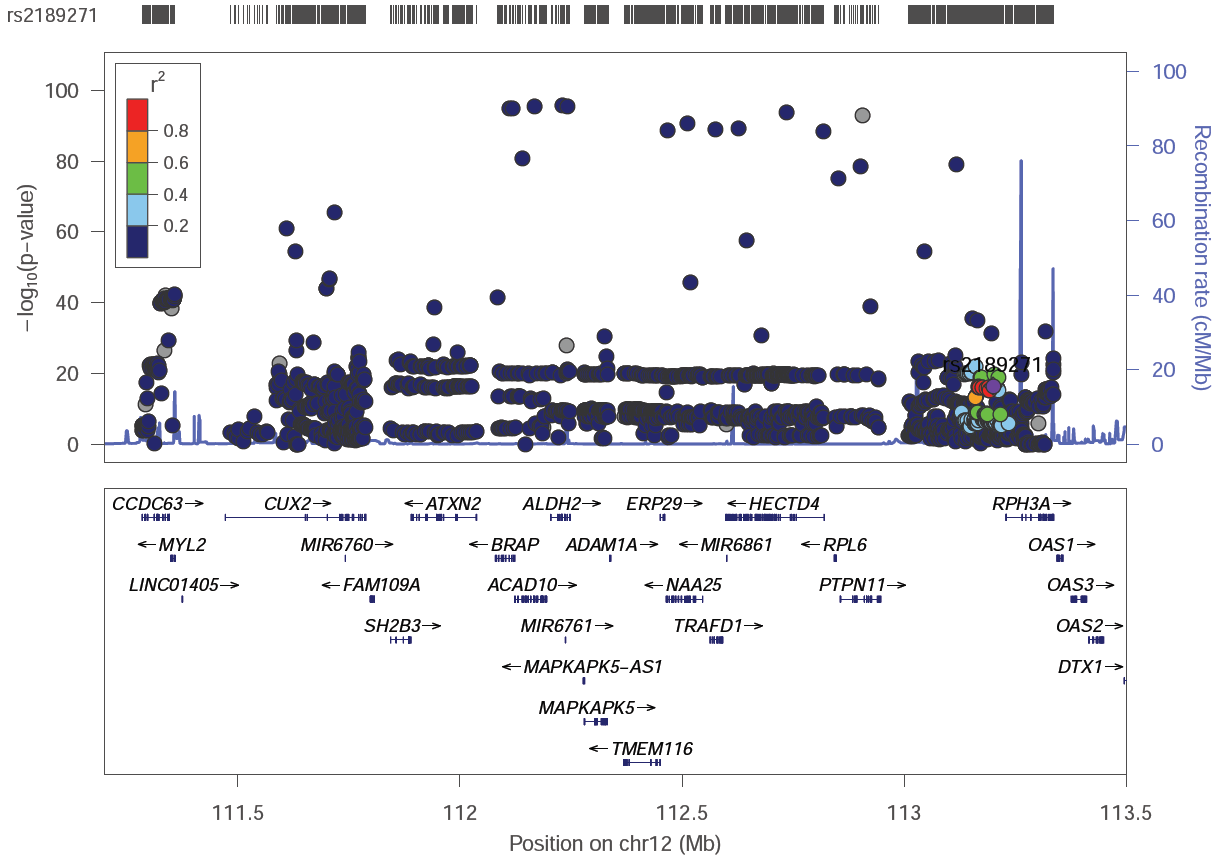


**Supplementary Fig. S4.** Linkage disequilibrium in a candidate region for excessive alcohol consumption around *CUX2*, *BRAP*, and *ALDH2*.Linkage disequilibrium was measured by r^2^. The total distance represented was 493 kb. For rs3782886 (*BRAP*) and rs671 (*ALDH2*), r^2^ was 0.98. For rs7398833 (*CUX2*) and rs671 (*ALDH2*), r^2^ was 0.02.


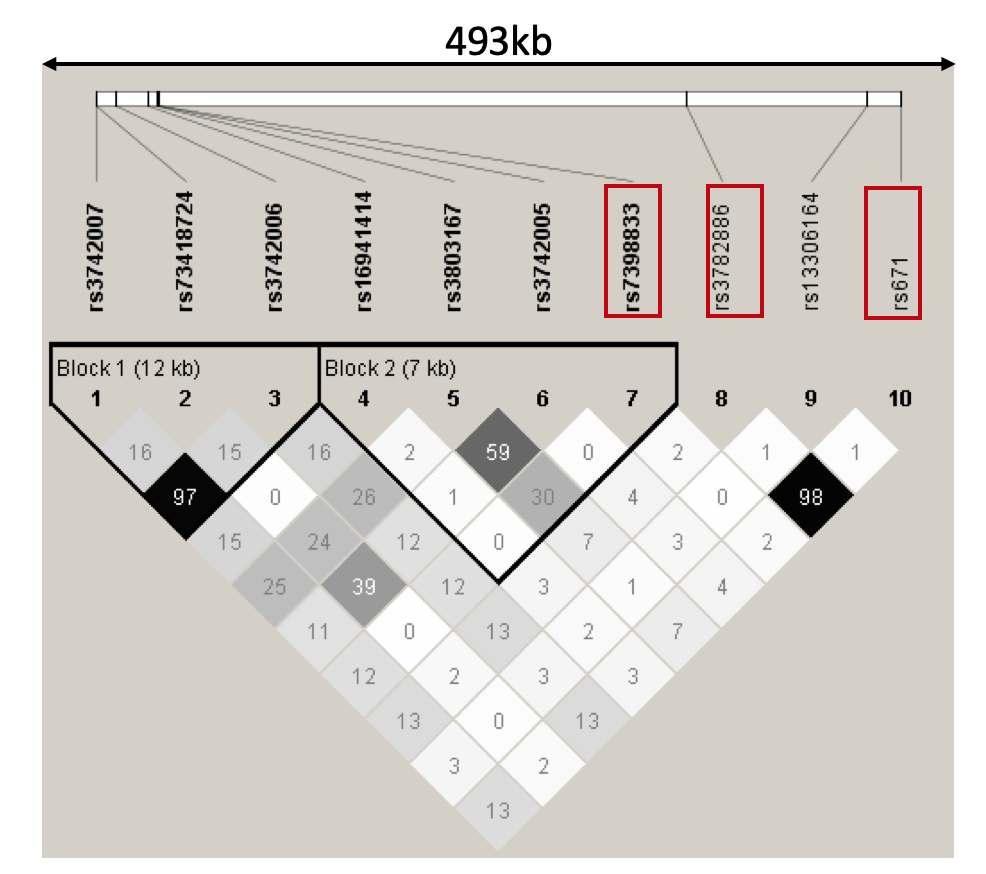


**Supplementary Fig. S5.** Closely related domains of phenotypes for rs671 (*ALDH2*) and rs3782886 (*BRAP*) in the PheWAS.


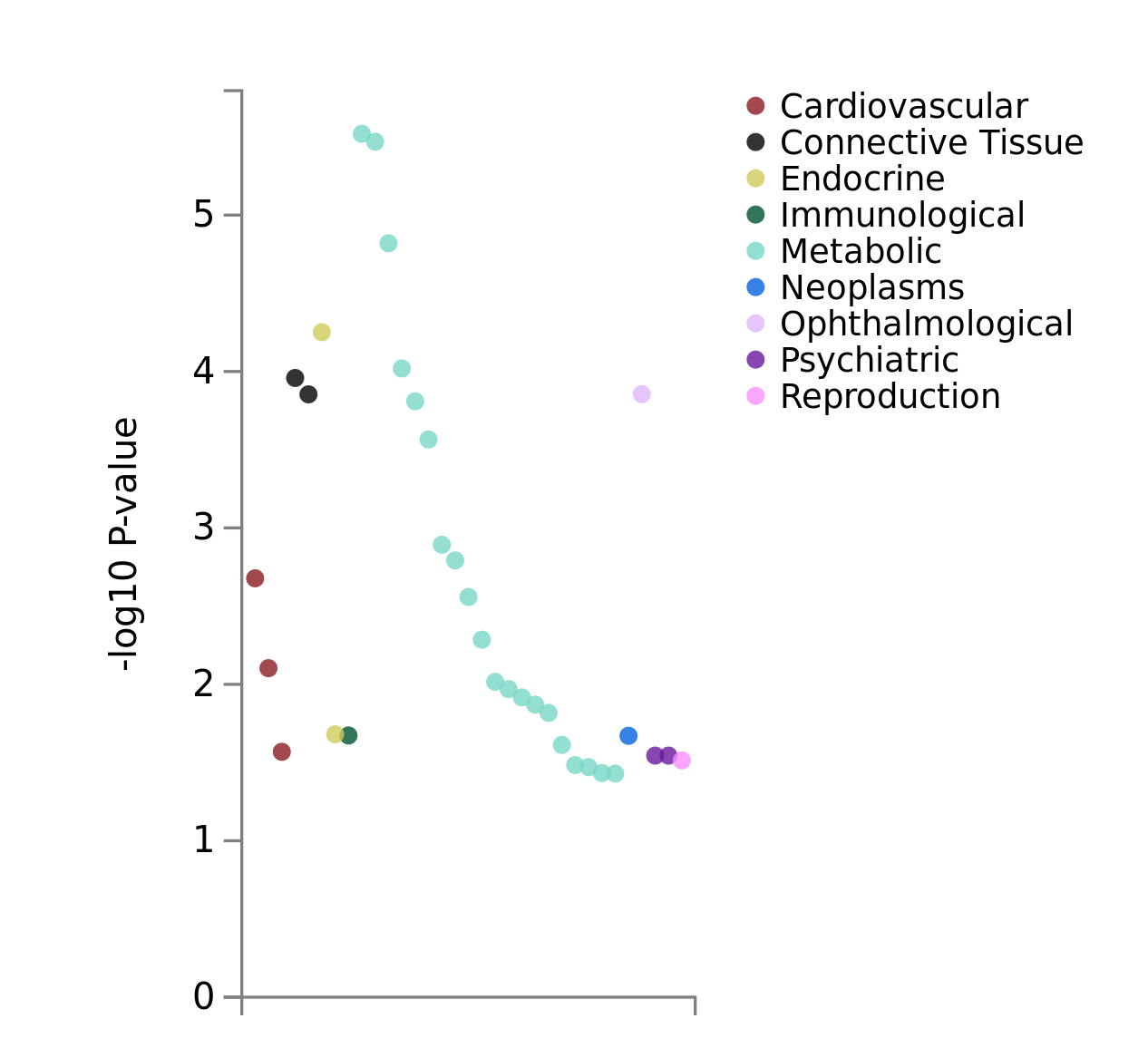


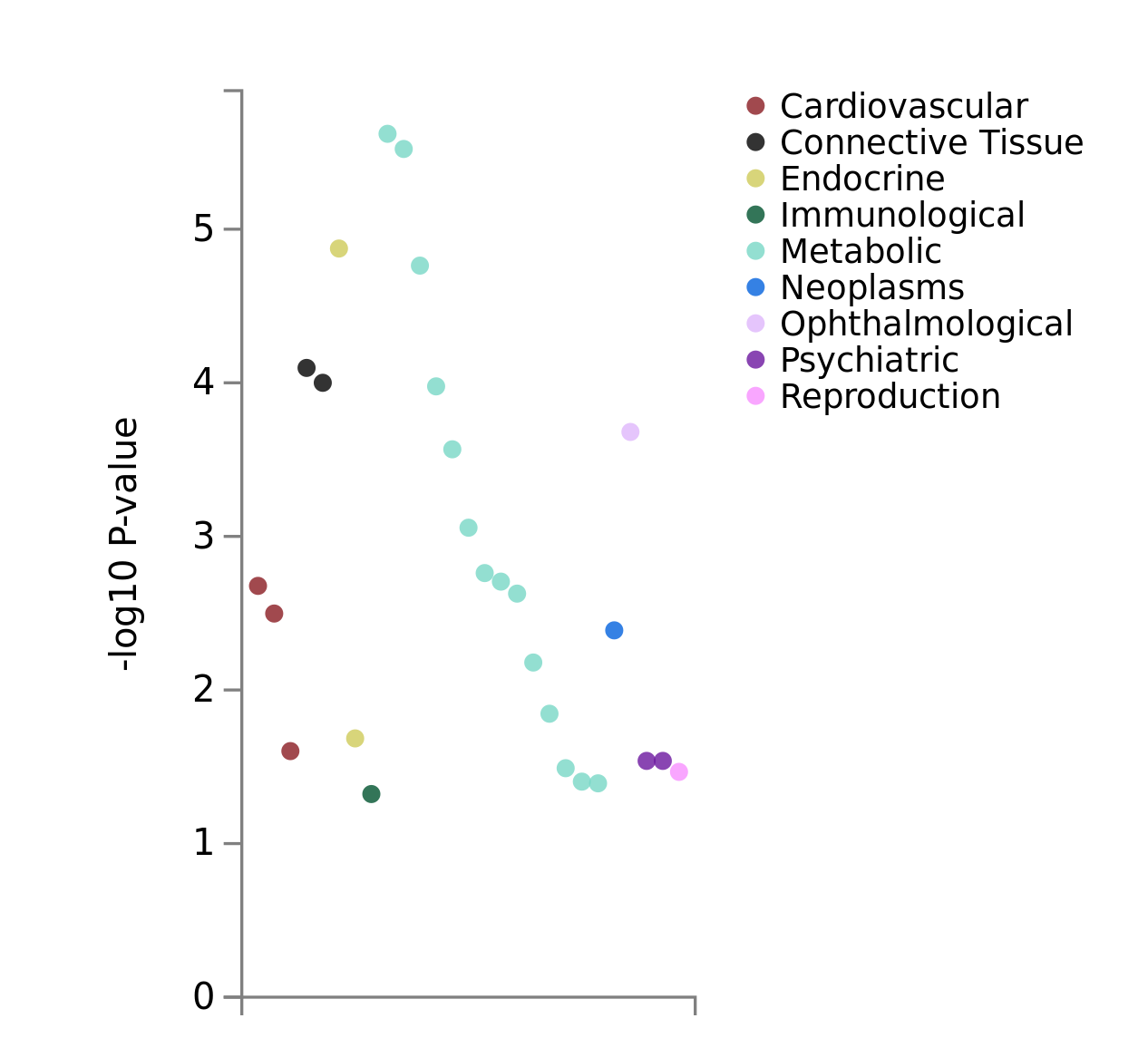


**Supplementary Fig. S6.** (a) The distributions of γ-GT levels across various regions. (b) The prevalence of excessive alcohol consumption is likely different among individuals from northern, central, southern and eastern Taiwan. Those on the outlying islands had higher frequencies of excessive alcohol consumption.

(a)


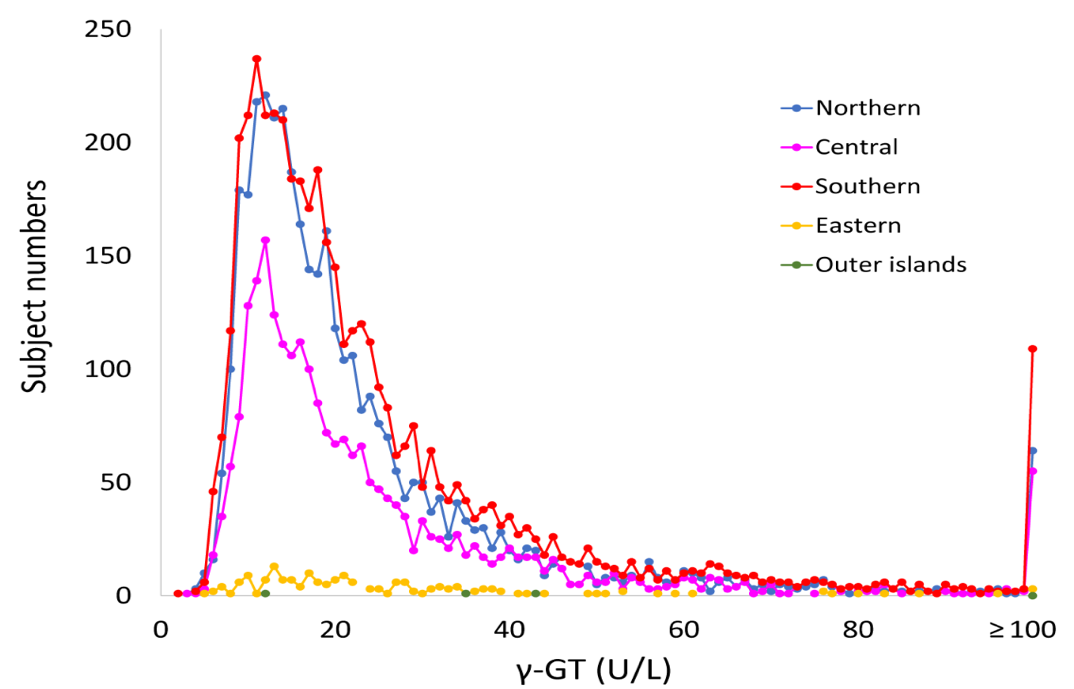


(b)


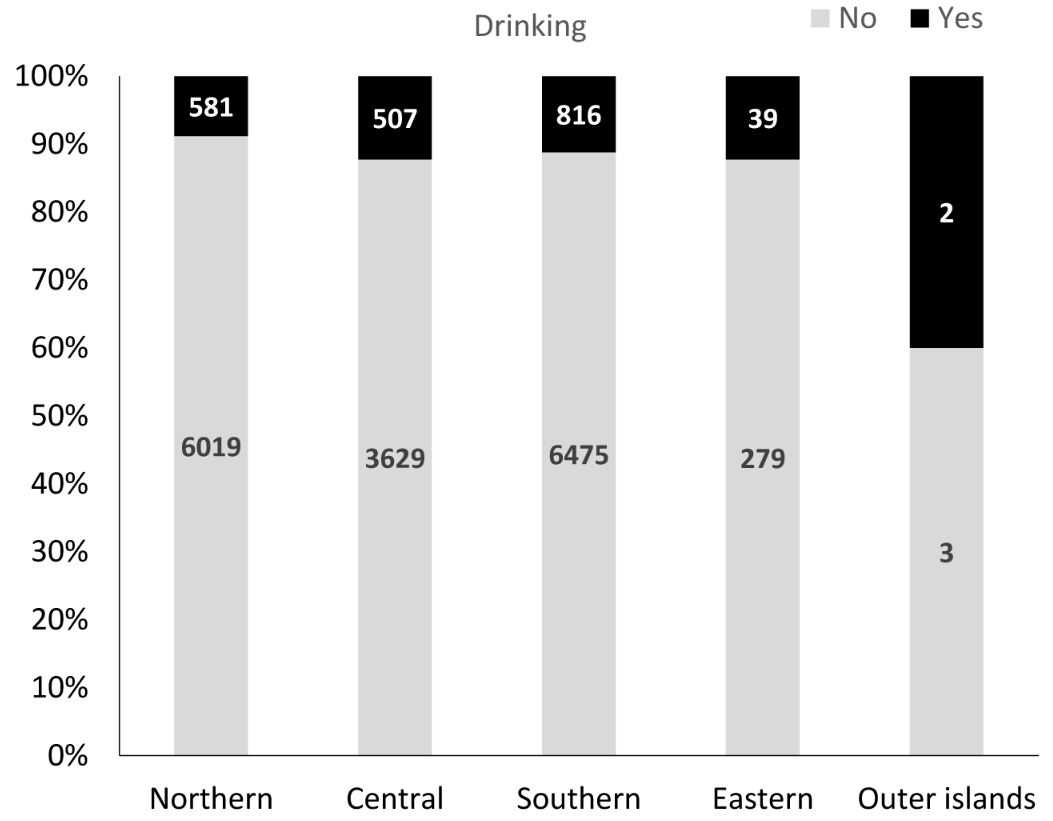


Excessive alcohol consumption

**Supplementary Table S1.** Logistic regression models of associations between alcohol consumption and 31 candidate genes on chromosome 12.

| Symbol | start | end | minimum *p* value of alcohol consumption | Number of total SNPs |
| --- | --- | --- | --- | --- |
| *ALDH2* | 112204691 | 112247782 | 1.73E-96 | 38 |
| *ACAD10* | 112123857 | 112194903 | 3.78E-96 | 39 |
| *BRAP* | 112079950 | 112123790 | 7.68E-96 | 25 |
| *HECTD4* | 112597992 | 112819896 | 1.19E-94 | 280 |
| *PTPN11* | 112856155 | 112947717 | 8.45E-94 | 37 |
| *NAA25* | 112464500 | 112546826 | 2.27E-91 | 94 |
| *TRAFD1* | 112563305 | 112591407 | 8.44E-90 | 22 |
| *RPH3A* | 113008184 | 113336686 | 6.35E-80 | 557 |
| *RPL6* | 112842994 | 112856642 | 5.46E-76 | 7 |
| *CUX2* | 111471828 | 111788358 | 2.76E-66 | 346 |
| *MYL2* | 111348623 | 111358526 | 1.05E-43 | 30 |
| *CCDC63* | 111284573 | 111345339 | 7.33E-43 | 99 |
| *ATXN2* | 111890018 | 112037480 | 2.60E-39 | 117 |
| *OAS1* | 113344582 | 113369990 | 2.38E-33 | 94 |
| *OAS3* | 113376157 | 113411054 | 1.55E-32 | 87 |
| *C12orf76* | 110465872 | 110511491 | 3.81E-32 | 57 |
| *MAPKAPK5* | 112279782 | 112334343 | 2.48E-31 | 121 |
| *TMEM116* | 112369086 | 112450970 | 1.35E-29 | 143 |
| *SH2B3* | 111843752 | 111889427 | 9.87E-25 | 24 |
| *FAM109A* | 111798455 | 111806925 | 1.01E-20 | 13 |
| *ERP29* | 112451120 | 112461255 | 3.54E-20 | 10 |
| *IFT81* | 110562140 | 110656602 | 2.47E-19 | 16 |
| *DTX1* | 113494514 | 113535833 | 9.05E-17 | 40 |
| *OAS2* | 113416200 | 113449528 | 1.40E-16 | 67 |
| *GIT2* | 110367607 | 110434194 | 1.00E-14 | 91 |
| *HVCN1* | 111065646 | 111142755 | 3.19E-14 | 11 |
| *PPTC7* | 110969120 | 111021125 | 5.29E-14 | 16 |
| *TCHP* | 110338069 | 110421646 | 9.32E-13 | 124 |
| *ANKRD13A* | 110436991 | 110477568 | 1.74E-12 | 46 |
| *TRPV4* | 110220890 | 110271212 | 4.71E-12 | 50 |
| *SDS* | 113830250 | 113864106 | 2.64E-08 | 23 |

Adjusted for age, gender, and 10 PCs.

**Supplementary Table S2.** Logistic and linear regression models of associations between *ALDH2*, excessive alcohol consumption, and γ-GT.

| SNP | Chromosome position | Location | Alcohol consumption | |  | γ-GT | |
| --- | --- | --- | --- | --- | --- | --- | --- |
|  |  |  | *p* value | FDR |  | *p* value | FDR |
| rs2283354 | 12:112206685 | Intron1 | 3.88E-10 | 5.30E-10 |  | 0.0005 | 0.001 |
| rs56884502 | 12:112207300 | Intron1 | 3.45E-10 | 5.30E-10 |  | 0.0005 | 0.001 |
| rs74849450 | 12:112208838 | Intron1 | 2.73E-10 | 4.94E-10 |  | 0.0004 | 0.0009 |
| rs2238152 | 12:112214459 | Intron1 | 3.78E-10 | 5.30E-10 |  | 0.0004 | 0.0009 |
| rs11613713 | 12:112217138 | Intron1 | 4.45E-10 | 5.64E-10 |  | 0.0004 | 0.0009 |
| rs11609628 | 12:112217208 | Intron1 | 4.03E-10 | 5.30E-10 |  | 0.0004 | 0.0009 |
| rs79463616 | 12:112221353 | Intron3 | 5.25E-10 | 6.05E-10 |  | 0.0004 | 0.0009 |
| rs4648328 | 12:112222788 | Intron3 | 5.25E-10 | 6.05E-10 |  | 0.0004 | 0.0009 |
| rs440 | 12:112228714 | Intron6 | 3.40E-10 | 5.30E-10 |  | 0.0004 | 0.0009 |
| rs441 | 12:112228849 | Intron6 | 2.20E-10 | 4.62E-10 |  | 0.0004 | 0.0009 |
| rs4646776 | 12:112230019 | Intron8 | 1.73E-96 | 4.36E-95 |  | 1.66E-06 | 3.58E-05 |
| rs4646777 | 12:112230036 | Intron8 | 2.07E-10 | 4.62E-10 |  | 0.0004 | 0.0009 |
| rs1362197604 | 12:112231389 | Intron9 | 3.61E-10 | 5.30E-10 |  | 0.0004 | 0.0009 |
| rs78783055 | 12:112233495 | Intron9 | 2.31E-10 | 4.62E-10 |  | 0.0004 | 0.0009 |
| rs4646778 | 12:112235783 | Intron9 | 2.72E-10 | 4.94E-10 |  | 0.0004 | 0.0009 |
| rs75480510 | 12:112240192 | Intron11 | 2.13E-10 | 4.62E-10 |  | 0.0003 | 0.0009 |
| rs11066025 | 12:112240696 | Intron11 | 7.58E-29 | 9.60E-28 |  | 0.010 | 0.019 |
| rs671 | 12:112241766 | Exon12 (missense Glu ⇒ Lys G ⇒ A) | 2.30E-96 | 4.36E-95 |  | 1.89E-06 | 3.58E-05 |
| rs77422707 | 12:112244053 | Intron12 | 4.05E-10 | 5.30E-10 |  | 0.0003 | 0.0009 |
| 12:112244140 | 12:112244140 | Intron12 | 6.13E-10 | 6.85E-10 |  | 0.0002 | 0.0009 |

Adjusted for age, gender, and 10 PCs; FDR, False Discovery Rate;

Location based on the ALDH2 isoform NM_000690.3.

**Supplementary Table S3.** Conditional analysis of the independent signals among rs671 (*ALDH2*) and rs3782886 (*BRAP*).

| Parameter | Regression coefficient | 95% Confidence Interval | Sum of Squared Error | *p* value of Partial F-test |
| --- | --- | --- | --- | --- |
| Model 1:$\gamma$-GT=sex + age + 10 principal components + rs671 | | | 13324296 |  |
| rs671 | 4.64 | (2.16, 7.12) |  |  |
|  |  |  |  |  |
| Model 2:$\gamma$-GT =sex + age + 10 principal components + rs3782886 | | | 13324086 |  |
| rs3782886 | -2.63 | (-4.00, -1.30) |  |  |
|  |  |  |  |  |
| Model 3:$\gamma$-GT = sex + age + 10 principal components + rs671 + rs3782886 | | | 13314402 |  |
| rs671 | 3.54 | (1.07,6.02) |  |  |
| rs3782886 | -2.02 | (-3.41, -0.62) |  |  |
|  |  |  |  |  |
| Model 4:$\gamma$-GT =sex + age + 10 principal components + rs7298833 NA | | | | |
| rs7298833 | -4.84 | (-14.71, 5.02) |  |  |
|  |  |  |  |  |
| Model 5:$\gamma$-GT =sex + age + 10 principal components + rs671 + rs3782886 + rs7298833 NA | | | | |
| rs671 | 3.54 | (1.06,6.02) |  |  |
| rs3782886 | -1.98 | (-3.38, -0.59) |  |  |
| rs7298833 | -3.64 | (-13.51, 6.24) |  |  |
|  |  |  |  |  |
| Model 1 - Model 3 |  |  |  | <0.01 |
|  |  |  |  |  |
| Model 2 - Model 3 |  |  |  | <0.01 |

Model 1, Model 2 and Model 3 used a multivariate regression model.

NA: rs7398833 (*CUX2*) was not included in the conditional analysis because not statistically significant in Model 4 and Model 5.
